# Extended Epidemiological Models for Weak Economic Region: Case Studies of the Spreading of COVID-19 in the South Asian Subcontinental Countries

**DOI:** 10.1155/2021/7787624

**Published:** 2021-10-19

**Authors:** Shafayat Bin Shabbir Mugdha, Mahtab Uddin, Md. Toriqul Islam

**Affiliations:** ^1^Department of Computer Science & Engineering, United International University, Dhaka 1212, Bangladesh; ^2^Institute of Natural Sciences, United International University, Dhaka 1212, Bangladesh; ^3^Department of Mathematics, Bangladesh University of Engineering & Technology, Dhaka 1000, Bangladesh

## Abstract

The ascendancy of coronavirus has become widespread all around the world. For the prevention of viral transmission, the pattern of disease is explored. Epidemiological modeling is a vital component of the research. These models assist in studying various aspects of infectious diseases, such as death, recovery, and infection rates. Coronavirus trends across several countries may analyze sufficiently using SIR, SEIR, and SIQR models. Across this study, we propose two modified versions of the SEIRD method for evaluating the transmission of this infectious disease in the South Asian countries, more precisely, in the south Asian subcontinent. The SEIRD model is updated further by fusing some new factors, namely, isolation for the suspected people and recovery and death of the people who are not under the coverage of healthcare schemes or reluctant to receive treatment for various catastrophes. We will investigate the influences of those ingredients on public health-related issues. Finally, we will predict and display the infection scenario and relevant elements with the concluding remarks through the statistical analysis.

## 1. Introduction

The recently detected SARS-CoV-2 coronavirus has led to a widespread epidemic pandemic named COVID-19 [1]. In early 2020, after the December 2019 incident at the city Wuhan in China, the World Health Organization (WHO) identified SARS-CoV-2 being a substitution style of COVID. The explosion rapidly expanded around the world. More than 200 countries, including Bangladesh, are afterward evaluated with confirmed infections. This dangerous infection has been poisonous by and large. The rate began to rise exponentially with time all around the world.

As per the WHO, worldwide starting on 31 May 2020, there are 6,218,927 confirmed cases of COVID-19, including 372,344 deaths. Regardless of its fast transmission rate, national emergency response plans, public health efforts, and public guidelines have eased back its turn of events and decreased the size of the COVID-19 episode, forestalling a large number of cases within 50 days in China, South Korea, Taiwan, Vietnam, New Zealand, etc. [2, 3]. Strength (*S*), weakness (*W*), opportunity (*O*), and threat (*T*) investigation technique distinguishes an essential premise and surveys a significant and relative way to deal with, hinder, and control the COVID-19 pandemic [4].

Earlier in May, the WHO stated a requirement for authorities to abolish shut or restriction, with six rules: (i) spread of infection under restriction; (ii) detection, testing, insulation, treatment of all cases of infection, and follow-up of every case; (iii) dangers to susceptible spots, such as nursing homes, are minimized; (iv) defensive measures are set up for instructive organizations, working environments, and other fundamental spots; (v) the probable danger of imported new cases is overseen; and (vi) the networks are altogether taught, drew in, enabled, and willing to work in sync with the new norm or ordinary. There ought to be an essential examination to lift the lockdown or closure. Lifting the lockdown too soon or excessively fast can raise the speed of the infection [5].

Bangladesh, one of the South Asian countries, is affirmed the maiden COVID-19 case on 8 March 2020. During the COVID-19 epidemic, the Bangladeshi Government designated the “public leave” as the lockdown on 26 March and extended it into seven different time zones until 30 May 2020. As a result, the psychosocial and socioeconomic crisis of this country started to decrease [6]. In the first sociomonetary weak assemblages, the two words “public leave” and “lockdown” produced misunderstanding. As of now, Bangladesh goes through far and wide local area transmission while the lockdown was removed on 30 May 2020. Starting on 31 May 2020, after the 65 days of lockdown, Dhaka was typical as was regular where no friendly removal or any well-being rule was kept up.

Many researchers from many countries have tried to estimate a standard epidemiological model of coronavirus situations based on SIS, SIR, SIRD, SEIR, SIQR, and SEIRD models, etc. The epidemiological model is appropriated to design and evaluate procedures to prevent infection and as a manual for the performance of patients in whom the disease has effectively evolved [7, 8].

A huge part of the mathematical research of the spread of overwhelming ailments starts from the classic compartmental models of Kermack and McKendrick (1927 and 1932) [9, 10]. These models partition the population into a few distinct compartments and determine how specialists get across the different compartments after some time. The SIRD epidemic model that Acemoglu et al. and Fernández-Villaverde and Jones analyzed in their paper is one among those compartmental models [11, 12]. Hethcote presents a valuable outline of this class of models and a couple of their hypothetical properties; Morton and Wickwire showed the best approach to apply ideal control techniques to them [13, 14].

The SIR model provides a hypothetical structure to research its spread inside a local area. Cooper et al. proposed the susceptible, infected, and removed (SIR) model with the difference that a total population is not defined or kept consistent and the number of susceptible individuals does not decline equability [15]. It is hard to measure an exact parameter because the information is generally defective on account of a shortfall of detailing. Mathematical models even have abilities still as limitations. Rarely, the researcher will be able to locate a genuine mix of functional data that will lead to a solution. The spread of the COVID-19 model in some Asian and European countries has been analyzed by the experts [16, 17]. They required time-based parameters in which the SIR model is approximated, while COVID-19 adapts. Cumulative data for the Euclidean SIR network model is thoroughly rooted in the COVID-19 studies [18]. In [19], three measures of SIQS and SIQR models and six endemic models have been discussed for infectious diseases. According to the SIQR model, the original balance was found to be an unstable spiral with an adjusted incidence in the quarantine. Tiwari investigated on developments and the growth of cases in India, which cannot be avoided to better understand the evolution of COVID-19 in the world [20]. Another well-established model for COVID-19 evaluation is the SEIR model, which is discussed in [21]. The normal SEIR model epidemics are adapted to the precise dynamic and epidemic parameters of COVID-19 within the age-heterogeneous community [22].

**Figure 1:**
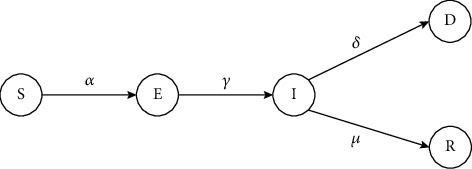
Basic SEIRD model.

The differential model of Fabiana Zama et al. for the analysis and predictive use of the Italian protozoan civil data issued February 24th, 2020, was proposed by the susceptible, infected, exposed, recovered, and dead (SEIRD) [23]. Since the Italian government imposed several restrictive measures at different times starting on 8 March 2020, a modification of the SEIRD model is proposed by introducing a time-based transmission rate. The SEIRD model is an epidemiological model and a variant of the SEIR high-level model [24, 25]. As shown in Figure 1, there are 5 fundamental factors in the classical SEIRD model, mainly, suspected (*S*), exposed (*E*), infected (*I*), died (*D*), and recovered (*R*). The suspected (*S*) category would be the group of people not infected but at risk. People prone to infection are in the category of exposed persons (*E*). In this category, people may not show any kind of COVID-19 symptoms for around 10-14 days. The set of people who have the symptoms of coronavirus and tested positive are in the infected (*I*) category, where the people dying and recovering are defined by *D*_*I*_ and *R*_*I*_, respectively. Following a brief period of recovery in which infected patients are healed, they are classified as recovered (*R*). Those that have not been treated are put in the group of those who have died (*D*).

In this study, we have worked with COVID-19 in Bangladesh based on the two modified versions of the SEIRD model, where one of them deals with the people who are not the coverage of the healthcare authority and the rest concerns about the people who are not willing to undergo the treatment due to several social and economic phenomena. By using these two models, we get a clear idea about the COVID-19 situation in Bangladesh. We have come up with some new information that will help us to predict the current situations. The primary goal of this work is to successfully predict the impact of COVID-19: (i) developing the desired models for predicting the impact of coronavirus, (ii) analyzing the number of infected persons, (iii) analyzing the impact of isolation factor, and (iv) analyzing death and recovery factor for the target communities. The factors mentioned above are the ingredients that are aimed at demonstrating in this research, and we will discuss these more elaborately in the next section.

## 2. Materials and Methods

In Bangladesh, many people do not get the scope to test for coronavirus or reach the healthcare centers for treatment at the proper time. They were later diagnosed with COVID-19 infection after they recovered or died; this group of people is classified as uncovered (*U*). Again, for the lack of social awareness, educational illiteracy, and financial scarcity of some people who are reluctant to take the treatment who are already exposed are classified as apathetic (*A*).

Here, we divide the exposed (*E*) people into two states, the first is isolated (*I*_*s*_), and the other is being infected (*I*). Isolated people are suspected and separated for possible infection but yet to be tested. The notations and symbols for the following models are defined in Table 1.

### 2.1. Model 1

**Figure 2:**
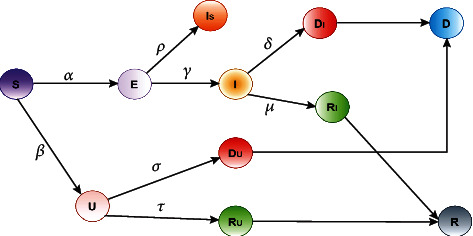
Flowchart of the Model 1.

In Model 1, we tend to outline that the suspect (*S*) of those who have not had COVID-19 however might be the risk from this state. We divided it into two states: one is exposed (*E*), and another one is uncovered (*U*). Then, from uncovered, we predict how many of them are recovering and how many are dying, which we have defined in the graph as *D*_*U*_ and *R*_*U*_. Next, we calculate the sum of *R*_*I*_ and *R*_*U*_ to predict the total number of recoveries which is *R*. Similarly, we can predict the total number of deaths which is *D* as the sum of *D*_*I*_ and *D*_*U*_. The flowchart of Model 1 is given in Figure 2.

Governing equations for the requirement of several physical and logical aspects, the mathematical formulation for the proposed Model 1 can be written by the system of governing equations as follows:

(1) $$\begin{array}{c} ∆S=-\alpha \left(\frac{S\times E}{N}\right)-\beta \left(\frac{S\times U}{N}\right), \end{array}$$

(2) $$\begin{array}{c} ∆E=\alpha \left(\frac{S\times E}{N}\right)-\gamma I-\rho I_{s}, \end{array}$$

(3) $$\begin{array}{c} ∆U=\beta \left(\frac{S\times U}{N}\right)-\sigma U-\tau U, \end{array}$$

(4) $$\begin{array}{c} ∆I=\gamma I-\delta I-\mu I, \end{array}$$

(5) $$\begin{array}{c} ∆I_{s}=\rho I_{s}, \end{array}$$

(6) $$\begin{array}{c} ∆D_{I}=\delta I, \end{array}$$

(7) $$\begin{array}{c} ∆R_{I}=\mu I, \end{array}$$

(8) $$\begin{array}{c} ∆D_{U}=\sigma U, \end{array}$$

(9) $$\begin{array}{c} ∆R_{U}=\tau U. \end{array}$$

By solving equations (1) to (9), we have the following constants representing various rate parameters.

(10) $$\begin{array}{c} \tau =\frac{∆R_{U}}{U},\\ \sigma =\frac{∆D_{U}}{U},\\ \mu =\frac{∆R_{I}}{I},\\ \delta =\frac{∆D_{I}}{I},\\ \rho =\frac{∆I_{s}}{I_{s}},\\ \gamma =\frac{∆I+∆D_{I}+∆R_{I}}{I},\\ \beta =\left(\frac{∆U+∆D_{U}+∆R_{U}}{S\times U}\right)\times N,\\ \alpha =\left(\frac{∆E+∆I+∆D_{I}+∆R_{I}+∆I_{s}}{S\times E}\right)\times N. \end{array}$$

### 2.2. Model 2

**Figure 3:**
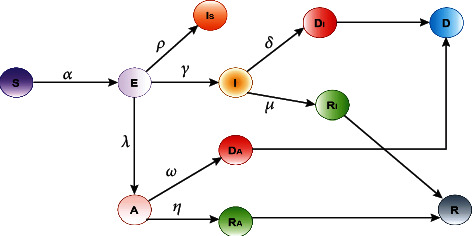
Flowchart of the Model 2.

In Model 2, we modify the original SEIRD model and designed a bit different from Model 1. We have divided exposed (*E*) people into three different states which are the apathetic (*A*), isolated (*I*_*s*_), and infected (*I*). Then, from apathetic, we predict how many of them are recovering and how many are dying, which we have defined in the graph as *D*_*A*_ and *R*_*A*_. Next, we calculate the sum of *R*_*I*_ and *R*_*A*_ to predict the total number of recoveries which is *R*. Similarly, we can predict the total number of deaths which is *D* as the sum of *D*_*I*_ and *D*_*A*_. The flowchart of Model 2 is given in Figure 3.

In a similar manner to the proposed Model 1, the mathematical formulation for the proposed Model 2 can be written as

(11) $$\begin{array}{c} ∆S=-\alpha \left(\frac{S\times E}{N}\right), \end{array}$$

(12) $$\begin{array}{c} ∆E=\alpha \left(\frac{S\times E}{N}\right)-\lambda A-\gamma I-\rho I_{s}, \end{array}$$

(13) $$\begin{array}{c} ∆A=\lambda A-\omega A-\eta A, \end{array}$$

(14) $$\begin{array}{c} ∆I=\gamma I-\delta I-\mu I, \end{array}$$

(15) $$\begin{array}{c} ∆I_{s}=\rho I_{s}, \end{array}$$

(16) $$\begin{array}{c} ∆D_{I}=\delta I, \end{array}$$

(17) $$\begin{array}{c} ∆R_{I}=\mu I, \end{array}$$

(18) $$\begin{array}{c} ∆D_{A}=\omega A, \end{array}$$

(19) $$\begin{array}{c} ∆R_{A}=\eta A. \end{array}$$

Previously, by solving equations (11) to (19), we have the following:

(20) $$\begin{array}{c} \eta =\frac{∆R_{A}}{A},\\ \omega =\frac{∆D_{A}}{A},\\ \mu =\frac{∆R_{I}}{I},\\ \delta =\frac{∆D_{I}}{I},\\ \rho =\frac{∆I_{s}}{I_{s}},\\ \gamma =\frac{∆I+∆D_{I}+∆R_{I}}{I},\\ \lambda =\frac{∆A+∆D_{A}+∆R_{A}}{A},\\ \alpha =\left(\frac{∆E+∆I+∆D_{I}+∆R_{I}+∆A+∆D_{A}+∆R_{A}+∆I_{s}}{S\times E}\right)\times N. \end{array}$$

## 3. Results and Discussions

We used extensions of the SEIRD model analysis to evaluate COVID-19 progression in Bangladesh, with the acceptable variations in practical purposes for the following parameters: infections (*I*), recoveries (*R*), and deaths (*D*), as well as the initial number of susceptible individuals (*S*). We present the validity of the proposed models with empirical results. We justify the accuracy of the predicted data as compared to real-time data collected from several reliable sources.

### 3.1. Data Set

Our main objective in this research is to predict the impact of the COVID-19 pandemic in Bangladesh. To do this, we need to know more real-time data about the current situation. This is the main troubleshot for this study. We have collected target data from different sources, such as news portals, health bulletins, and government/nongovernment agencies. Most of the time, we cannot ensure the authenticity of conventional news sources. So, we have worked to collect data from multiple reliable websites that provide real-time data, such as WHO, Worldometer, and IEDCR. We mainly collected data daily from the website Worldometer over the target period and gathered them as the required data set.

The data set includes the daily number of deaths, recoveries, tested cases, confirmed cases, number of infected people, uncovered/apathetic cases (collected locally), the number of people going into isolation, and so on. The data of 36 days from 24th March to 29th April of 2020 have been obtained.

Since the programs supporting the proposed methods can be well-trained for at most 15 days, we used the real-time data from 14th April to 29th April of 2020 to get the predicted data from 30th April to 14th May of 2020. In this case, our model learns the data from every day and then predicts the outcome of the next day. For example, on April 15, we again predict the values of April 16. Based on April 16, we predict the data of April 17, etc. That is how we can perfectly predict the results.

### 3.2. Graphical Representation

For validating the accuracy and adaptability of the proposed models, we investigate the graphical comparison between real-time data and predicted data for all of the attributes considered in this work.

Until May 14, the evolution of the COVID-19 in Bangladesh was compared to the real-time data. It is observed that the number of COVID-19-infected people in Bangladesh is exponentially increased from 24th March 2020.

**Figure 4:**
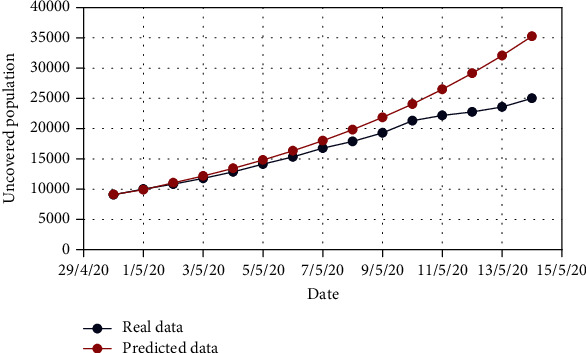
Comparison of the uncovered population for Model 1.

**Figure 5:**
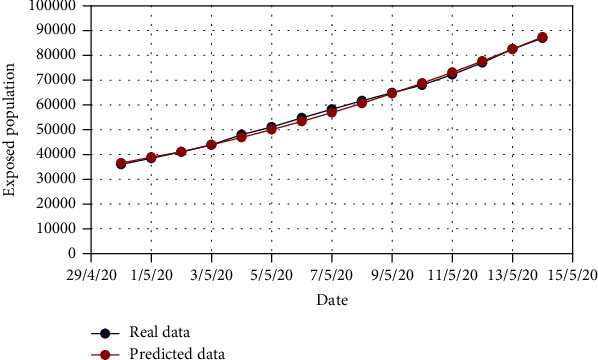
Comparison of the exposed population for Model 1.

**Figure 6:**
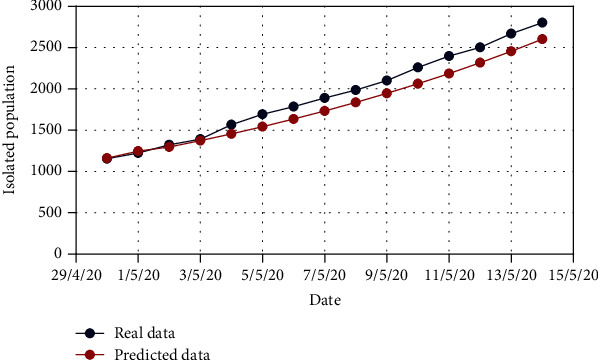
Comparison of the isolated population for Model 1.

**Figure 7:**
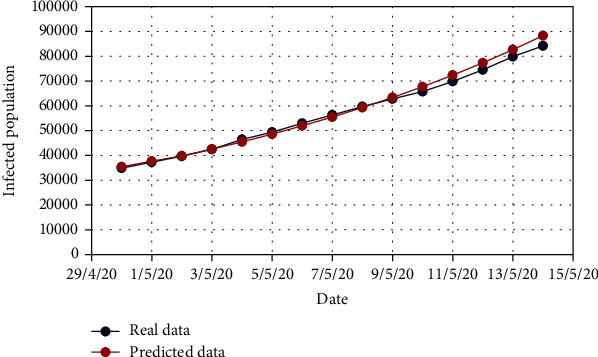
Comparison of the infected population for Model 1.

**Figure 8:**
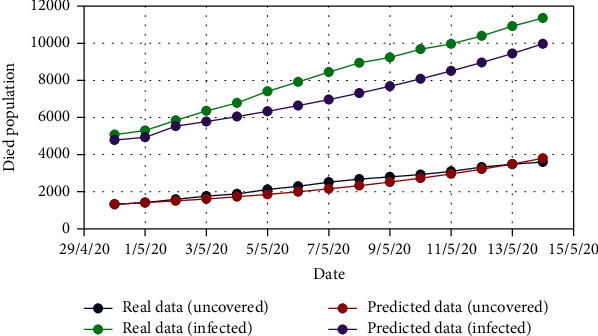
Comparison of the died population for Model 1.

**Figure 9:**
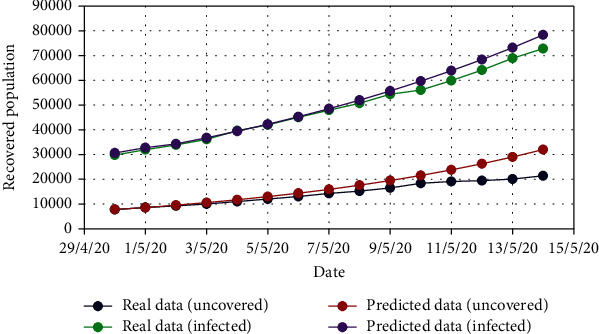
Comparison of the recovered population for Model 1.

For Model 1, Figures 4 and 5 show the variation in the uncovered (*U*) and exposed population (*E*) for both real-time and predicted data, whereas Figures 6, 7, 8, and 9 depict the isolated (*I*_*s*_), infected (*I*), died (*D*), and recovered (*R*) individuals, respectively, for those data.

**Figure 10:**
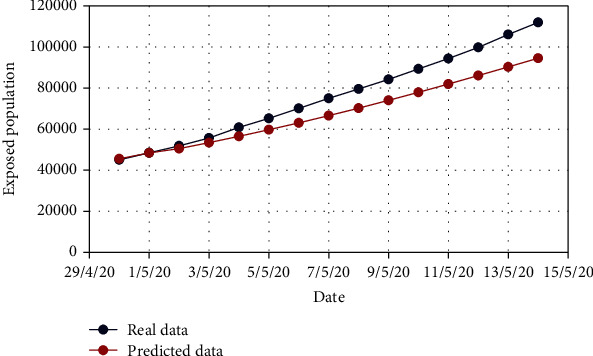
Comparison of the exposed population for Model 2.

**Figure 11:**
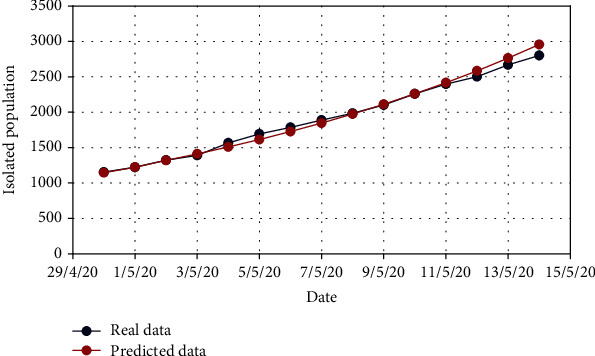
Comparison of the isolated population for Model 2.

**Figure 12:**
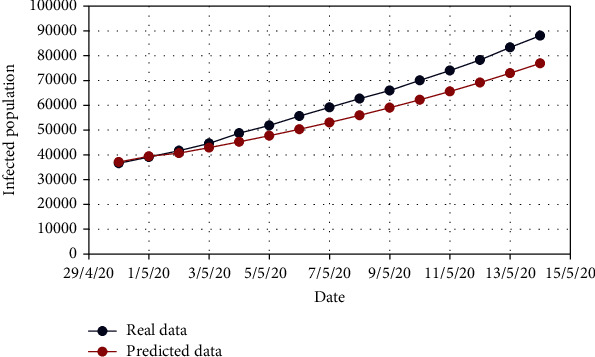
Comparison of the infected population for Model 2.

**Figure 13:**
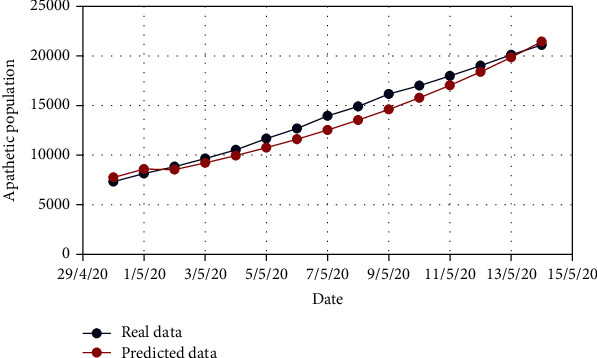
Comparison of the apathetic population for Model 2.

**Figure 14:**
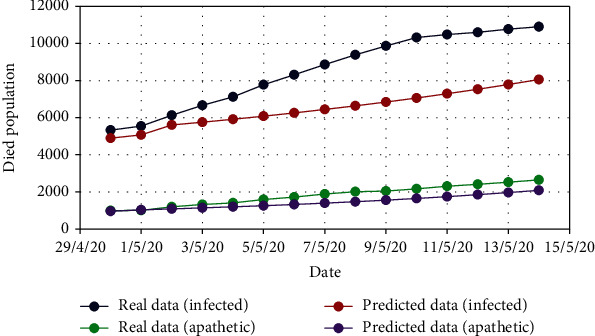
Comparison of the died population for Model 2.

**Figure 15:**
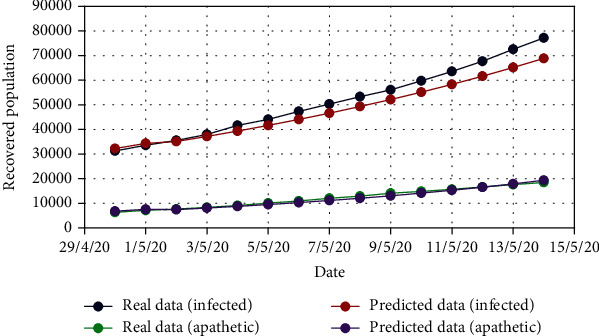
Comparison of the recovered population for the Model 2.

In the similar manner, for Model 2, Figure 10 displays the change in the exposed population (*E*) for both real-time and predicted data, whereas Figures 11, 12, 13, 14, and 15 illustrate the isolated (*I*_*s*_), infected (*I*), apathetic (*A*), died (*D*), and recovered (*R*) individuals, respectively, for the target data.

In all of the figures, we have exhibited the comparison between the real-time data and the predicted data. Also, we have compared the death and recovery for both uncovered and infected people in Model 1, whereas we have done the same for infected and apathetic people in Model 2. A common trend has been observed in each of the comparative graphs for the real-time data and the predicted data in both of the models that we have proposed. So, we can claim that the proposed extensions of the SEIRD model are compatible with the data prediction approaches and can efficiently apply to the practical events of epidemiology.

### 3.3. Statistical Analysis

In this section, we will have some statistical discussion on the predicted data. We will discuss the correlation and regression analysis of the death and recovery for the proposed methods. For numerical computation, programming through Microsoft Excel is used.

For Model 1, the correlation coefficient of people who died from uncovered and infected is 0.9902, and regression coefficients are 0.3375 and 2.9054, for uncovered dominant and infected dominant, respectively. Again, the correlation coefficient of people recovered from uncovered and infected is 0.9881, and regression coefficients are 0.3662 and 2.6665, for uncovered dominant and infected dominant, respectively. For Model 2, the correlation coefficient of people who died from apathetic and infected is 0.9919, and regression coefficients are 0.2286 and 4.3028, for apathetic dominant and infected dominant, respectively. Again, the correlation coefficient of people recovered from infected and apathetic is 0.9934, and regression coefficients are 0.2564 and 3.8494, for apathetic dominant and infected dominant, respectively.

From the above statistical measures, it is evident that the death/recovery of the people who are uncovered or apathetic are fully correlated with the infected people. Also, in case of the dependency of the death/recovery of the people, infected people are highly dominant over the other people.

## 4. Conclusion

In this research, two extensions of the SEIRD model were formed that had a different architecture than the classical SEIRD model with some new aspects, for instance, isolated, uncovered, and apathetic people with the measurement of death and recovery. Based on these new characteristics, we have investigated the propagation in Bangladesh of the COVID-19. We have reached satisfactory outcomes for the two abovementioned extensions. We have witnessed that the predicted results have a very identical trend to that of real-time data. From the statistical analysis, it is evident that the number of uncovered/apathetic people is strictly correlated with the infected people in both of the proposed models. In addition, it is observed that the infected people are dominant in the cases of deaths and recoveries.

From the analysis of the real-time data and the predicted data, we can come to a conclusion that the deaths of uncovered people are very few in comparison to the infected people. This incident encourages illiterate people to be apathetic to the current treatment. Moreover, the high recovery rate of uncovered people is another catalyst of the apathy of taking healthcare services.

In the future, we will study the effects of more epidemiological diseases utilizing the proposed models. We would like to employ the machine learning algorithms to the models to enhance a better efficiency and to achieve a more promising outcome.

## Figures and Tables

**Table 1:** List of parameters.

Symbol	Name of the parameter
*S*	Susceptible population
*E*	Exposed population
*I*	Infected population
*D*_*I*_	Died population from infection
*R*_*I*_	Recovered population from infection
*I*_*s*_	Isolated population
*U*	Uncovered population
*D*_*U*_	Died population from uncovered
*R*_*U*_	Recovered population from uncovered
*A*	Apathetic population
*D*_*A*_	Died population from apathetic
*R*_*A*_	Recovered population from apathetic
*α*	Rate of exposed population
*β*	Rate of uncovered population
*λ*	Rate of apathetic population
*ρ*	Rate of isolated population
*γ*	Rate of infected population
*δ*	Rate of infected population death
*μ*	Rate of infected population recovery
*σ*	Rate of uncovered population death
*τ*	Rate of uncovered population recovery
*ω*	Rate of apathetic population death
*η*	Rate of apathetic population recovery

## Data Availability

The data and codes are available at the following link: https://github.com/Shafayat-Mugdha/Covid-19_Research_Work?fbclid=IwAR2UwUSyqdm8iYalNrzgurEvauccophnHMHvHCDUflXaBaEmoG9btMLgV_4.

## Additional Points

*WHO*. https://www.who.int. *Worldometer*. https://www.worldometers.info; *IEDCR*. https://www.iedcr.org.
