# Corrigendum to “Extended Epidemiological Models for Weak Economic Region: Case Studies of the Spreading of COVID‐19 in the South Asian Subcontinental Countries”

**DOI:** 10.1155/bmri/9782879

**Published:** 2025-09-19

S. B. S. Mugdha, M. Uddin, and M. T. Islam, “Extended Epidemiological Models for Weak Economic Region: Case Studies of the Spreading of COVID‐19 in the South Asian Subcontinental Countries,” *BioMed Research International* 2021 (2021): 7787624, https://doi.org/10.1155/2021/7787624.

In the article, the name of the parameter for *N* in Table 1 is omitted. The correct Table 1 is shown as follows:

**Table 1:** List of parameters.

**Symbol**	**Name of the parameter**
*N*	Total population
*S*	Susceptible population
*E*	Exposed population
*I*	Infected population
*D*_*I*_	Dead population from infection
*R*_*I*_	Recovered population from infection
*I*_*s*_	Isolated population
*U*	Uncovered population
*D*_*U*_	Dead population from uncovered
*R*_*U*_	Recovered population from uncovered
*A*	Apathetic population
*D*_*A*_	Dead population from apathetic
*R*_*A*_	Recovered population from apathetic
*α*	Rate of exposed population
*β*	Rate of uncovered population
*λ*	Rate of apathetic population
*ρ*	Rate of isolated population
*γ*	Rate of infected population
*δ*	Rate of infected population death
*μ*	Rate of infected population recovery
*σ*	Rate of uncovered population death
*τ*	Rate of uncovered population recovery
*ω*	Rate of apathetic population death
*η*	Rate of apathetic population recovery

We apologize for this error.

